# TCR/pMHC Interaction: Phenotypic Model for an Unsolved Enigma

**DOI:** 10.3389/fimmu.2016.00467

**Published:** 2016-11-09

**Authors:** Jesús Gálvez, Juan J. Gálvez, Pilar García-Peñarrubia

**Affiliations:** ^1^Department of Physical Chemistry, Faculty of Chemistry, University of Murcia, Murcia, Spain; ^2^Department of Information and Communications Engineering, Computer Science Faculty, University of Murcia, Murcia, Spain; ^3^Department of Biochemistry and Molecular Biology B and Immunology, School of Medicine, University of Murcia, Murcia, Spain

**Keywords:** TCR activation, kinetic proofreading, stabilization and destabilization of TCR/pMHC complexes, dissociation and propagation rate constants, activation chain

## Abstract

TCR–pMHC interaction is the keystone of the adaptive immune response. This process exhibits an impressive capacity of speed, sensitivity, and discrimination that allows detecting foreign pMHCs at very low concentration among much more abundant self-pMHC ligands. However, and despite over three decades of intensive research, the mechanisms by which this remarkable discrimination and sensitivity is attained remain controversial. In kinetic proofreading mechanisms (KPR), an increase of specificity occurs by reducing the sensitivity. To overcome this difficulty, more elaborate models including feedback processes or induced rebinding have been incorporated into the KPR scheme. Here a new approach based on the assumption that the proofreading chain behaves differently for foreign- and self-pMHC complexes has been integrated into a phenotypic model in which the complexes responsible for T cell activation stabilize (for foreign peptides) or weaken (for foreign peptides), resulting in a dramatic increase in sensitivity and specificity. Stabilization and destabilization of complexes may be caused by conformational changes, rebinding, or any other process leading to variations in the dissociation rate constants of the complexes transmitting the activation. The numerical solution and the analytical expression for the steady-state response as a function of *k*_off_(*i*) (*i* = 0, 1, …, *N*, where *C*_0_, *C*_1_, …, *C_N_* are the complexes in the proofreading chain) are provided. The activation chain speeds up, and larger increases in sensitivity and discrimination are obtained if the rate of activation along the proofreading chain increases for foreign pMHCs and decreases for self-ligands. Experimental implications and comparison with current models are discussed.

## 1 Introduction

TCR–pMHC interaction leading to T cell activation is the keystone of the adaptive immune responses to infections and cancer and plays a decisive role in allergy, autoimmunity, and transplant rejection (1). The clonotypic receptor of T lymphocytes (T cell receptor, TCR) recognizes antigenic peptides accommodated in the groove of major histocompatibility complex (MHC) molecules expressed on the membrane of antigen-presenting cells (APC) or target cells (2). The engagement of TCR with its specific antigenic peptide (agonist)/MHC complex (pMHC) triggers intracellular signaling pathways that induce the expression of genes required for T cell-mediated effector functions, such as T cell proliferation, cytokine secretion, and cytotoxicity (3). While the repertoire of human T cells can recognize the enormous variety of antigenic peptides present in nature (4), only a small proportion of mature T cells can recognize a specific pMHC complex (5). T cells achievement of that property is accomplished by mean of a very high sensitive and specific pMHC recognition process whereby T cells are capable to respond quickly to very low levels of foreign pMHC but ignore huge amounts of self-pMHC. This process is called antigen discrimination, and it has been reported that even recognition of a single agonist pMHC can produce intracellular increases of Ca^+2^ (6) and cytolytic activity (7). In turn, selectivity is characterized by the ability of a particular TCR to discriminate between peptides differing in a single amino acid presented in a particular MHC allele. However intriguingly, mature peripheral T cells tolerate cells presenting only self-pMHC but are elicited by interaction with the same cells expressing even scarce quantities of foreign peptides. The successful outcome of these processes is critical because if discrimination fails, it leads to either infections or autoimmune diseases (8). In other words, the cell fate of the immune system relies on the capacity of T-cell signal transduction to satisfy the following three properties for appropriate initiation of the immune response: speed, sensitivity, and specificity (9).

The underlying mechanisms for these unique features of T cells function remain enigmatic, and different hypothesis, verbal, and theoretical models have been proposed along the past decades to explain T cell activation [reviewed by Zarnitsyna and Zhu (10) and Lever et al. (11)]. However, currently, and despite extensive experimental and theoretical work, there is no model relating the TCR–pMHC binding interaction to T cell activation that is consistent with the published experimental data (11).

By focusing on the three properties of T cell activation above mentioned, the simplest approach is the TCR occupancy model that is based on the requirement of a threshold for the number of TCR–pMHC bonds (12). This is supported by experimental observations of an increased stimulation level produced by increases of pMHC concentration and a density compensation for weaker ligands (13, 14). However, this model does not explain discrimination because high occupancy can be also attained for low-affinity pMHC by increasing its concentration. In addition, occupancy models have also been precluded by experiments showing that increases of low-affinity pMHC concentration do not activate T cells (15), while very low concentrations of a pMHC whose affinity is only threefold higher can actually do it (16).

Recent experimental and theoretical works (15, 17–20) suggest that the major influence in the discrimination process is exerted by the dissociation time of the TCR–pMHC complex. Thus, it has been pointed out (9, 21) that an ideal response in terms of specificity and sensitivity should imply the existence of a threshold time for a TCR–pMHC interaction (below which there is no T cell activation) and a number of ligand per cell as low as possible [ideally one single ligand, see Figure 1A in Ref. (9, 21)]. Interestingly, the kinetic proofreading (KPR) mechanism proposed by McKeithan (22) amplifies differences in affinities and dissociation times of pMHC ligands, which would permit discrimination among them. In this mechanism, pMHC ligands bind to TCRs to form a TCR–pMHC complex (*C*_0_) that goes through a sequence of *N* biochemical modifications (complexes *C*_1_, …, *C_N_*), which form the proofreading chain. Since in this chain only *C_N_* is the productive signaling complex, it introduces a delay in the activation transmission that must fulfill with the minimum threshold time required for successful signaling. However, it has been shown (9, 21) that although KPR can largely increase discrimination is at the expense of a large reduction in sensitivity. To overcome this difficulty, more elaborate models that include feedback processes or induced rebinding have been incorporated into the basic KPR scheme (9, 17, 21–26), although it has been suggested that the existence of a trade-off between sensitivity and specificity appears to be a general principle (24).

Based on these observations, we consider that differences between the dissociation times of the TCR–pMHC complexes formed among foreign and self-ligands are insufficient to explain the big discrepancies exhibited by T-cell activation induced by both kinds of ligands. Hence, we hypothesize that the proofreading chain leading to a productive response behaves quite differently for foreign and self-peptides, which causes that the resulting activation chains for both types of ligands have also different properties. This, in turn, would be the main factor responsible for their specific and distinct responses. Starting from this assumption we have developed a model where the complexes engaged in KPR stabilize (for foreign peptides) or weaken (for self-pMCH ligands), as the activation chain progresses, resulting in an enhanced response with a dramatic increase in sensitivity and specificity. In addition, further improvements in sensitivity and discrimination are obtained if the rate for activation propagation among the *C_i_* complexes increases for foreign ligands and decreases for self-complexes as activation progresses. The combination of these two effects reinforces and speeds up the transmission proofreading chain in the case of foreign peptides and delays and weakens (or even breaks down) the chain with self-ligands, which allows to explain why huge amounts of self-pMHCs are not able to activate T cells while, conversely, even a single foreign pMHC can trigger the T cell response.

## 2 Materials and Methods

### 2.1 Parameter Values

Number of TCRs *T_T_* = 2 × 10^4^; *k*_on_ = 5 × 10^−5^ s^−1^; *k_p_* = 1 s^−1^. There are no concentrations units: all concentrations in figures, in tables, and in rate constants are per cell. Thus, *k*_on_ = 5 × 10^−5^(molecule × s)^−1^; *k*_off_ _=_ 1/*τ* and *k_p_* represent in our model *k*_off_(0) and *k_p_*(0), respectively. These parameter values are similar to those used in Ref. (9, 11, 16). In addition to the above parameters, in the induced rebinding model [Dushek and van der Merwe (21)], the signaling decay rate (λ = 10^4^ s^−1^) and the rate of rebinding of the *C_i_* complexes *ρ_i_* are also required: *ρ_i_* = 10^3^ s^−1^ for *i* ≤ 21 increasing to 10^7^ s^−1^ for *i* = 25 [see Figure S2 in Ref. (21)].

### 2.2 Computations and Numerical Solution of the System of ODEs

**Figure 1:**
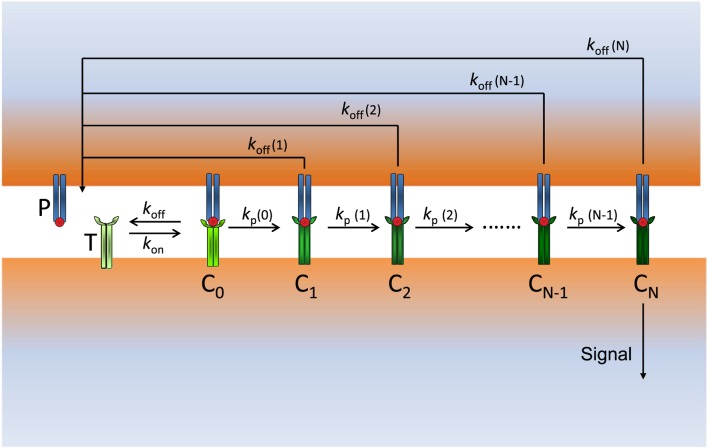
**Scheme of our modified KPR model: P (free pMHCs) and T (free TCRs) bind to form the complex pMHC–TCR (*C*_0_) with rate constants *k*_on_ and *k*_off_**. *C*_0_ initiates the proofreading chain that propagates activation through the complexes *C*_1_, *C*_2_, …, *C_N_*, where *C_N_* is the productive signaling complex at the end of the chain. The dissociation rate constants *k*_off_(*i*) (*i* = 0, 1, …, *N*) and the propagation rate constants *k_p_*(*i*) (*i* = 0, 1, …, *N − 1*) of the *C_i_* complexes change in a different way for foreign and self-ligands along the activation chain.

Our model (see Figure 1) is described by a system of ordinary differential equations (ODEs), which is given in Appendix. Numerical solution of the system of ODEs and all remaining calculations and plots were performed using *Mathematica 9.0*.

## 3 Results and Discussion

To maintain the model in Figure 1 as simple as possible, we add to the basic KPR scheme the hypothesis that stabilization/destabilization of the *C_i_* complexes and changes in the speed of activation propagation are due to variations in the values of the corresponding rate constants, *k*_off_(*i*) (*i* = 0, 1, …, *N*) and *k_p_*(*i*) (*i* = 0, 1, …, *N* − 1), which occur as proofreading progresses. Stabilization and destabilization of complexes may occur by feedback processes, conformational changes, rebinding, sinapse remodeling, or any other process leading to variations in the values of the dissociation and propagation rate constants of the complexes that transmit activation. In this context, it is worth mentioning that an elaborate experimental kinetic analysis of protein conformational changes recently published has shown that their conformational states have different rate constants and affinities (27). Further considerations and assumptions of our model are as follows:

- As in the classic KPR scheme, unbinding of pMHCs from *C_i_* complexes reverts the TCR to its initial unmodified state.
- Current models based on the KPR mechanism assume that the unbinding rate constant (*k*_off_ _=_ 1/*τ*) is the same for all the *C_i_* complexes and is equal to that of the first TCR–pMHC complex (*C*_0_) in the proofreading chain. However, this assumption is not justified because for *i > *0 these complexes go through a series of biochemical modifications whose actual values of *k*_off_ are unknown.
- The goal of T cell activation through TCR is to elicit efficient immune responses against cells presenting foreign peptides and ignore self-ligands. Hence, we assume that these different objectives should appear quantitatively reflected in the proofreading chain. One way to accomplish this goal is by considering that the values of *k*_off_ are not constant, but they vary as activation progresses so that the chain propagation is reinforced when an efficient immune response takes place (foreign peptide) and weakened or broken down with self-ligands.
- In our model, and for all kinds of peptides, *k*_off_ (0) (denoted as *k*_off_ for simplicity) is equal to 1/*τ* where *τ* is the dissociation time for the *C*_0_ complex. However, for *i* > 0, the values of *k*_off_(*i*) are unknown, although stabilization of the *C_i_* complexes would occur if *k*_off_(*i*) decreases along the chain propagation, while they are less stable if *k*_off_(i) increases as *i* → *N*. As we show below, this causes a dramatic enhancement in the capacity of discrimination and sensitivity between foreign and self-peptides regardless of whether there is little or no difference between their dissociation times.
- In the KPR and related models, the rate of propagation of the activation (*k_p_*) among the *C_i_* complexes is considered to have the same value along the proofreading chain. In our model, this assumption has been removed so that the values of *k_p_*(*i*) for foreign ligands increase to facilitate and speed up the productive signal as *i* → *N*, while for self-ligands those values decrease, which delays and weakens (or even breaks down) the activation chain. Quantitatively, the TCR–pMHC engagement time will be a function on *N* and the propagation rate constants for the *C_i_* complexes along the proofreading chain.
- Given that the experimental values of *k*_off_(i) and *k_p_*(i) are currently unknown, computational responses were obtained using several types of reasonable functions discussed in next sections to fulfill our assumptions for foreign and self-ligands.
- The reaction rate constants and other parameter values used for computation were similar to those used by François et al. (9), Altan-Bonnet and Germain (16), and Lever et al. (11) and are given in Materials and Methods.
- Responses from our model were computed by solving deterministic ordinary differential equations (ODEs), i.e., stochastic effects were not taken into account. Recent studies (9, 21) have shown a good agreement between deterministic and stochastic responses for this type of models.

### 3.1 Formulation of the Model

The detailed formulation of our model in terms of a system of ODEs is shown in Appendix. These equations must be solved numerically, and they allow us to obtain the response as the time activation progresses for given values of the number of TCR receptors and pMHC ligands and for any kind of *k*_off_(i) and *k_p_*(i) functions. In turn, the steady-state solution is derived by inserting the conditions *dC_i_*/*dt* = 0, *dP*/*dt* = 0 (or *dT*/*dt* = 0) (where *P* and *T* represent the number of unbound ligands and receptors) into the system of ODEs. Thus, the following analytical equations for all the *C_i_* complexes concentrations were obtained:

(1) $$C_{0}=\frac{C_{T}}{\mu };\;C_{i}=\gamma_{i}\;C_{0},\;1\leq i\leq N-1;\;C_{N}=\delta C_{T}$$

with

(2) $$\mu =1+\frac{k_{p}\left(N-1\right)}{k_{\text{off}}\left(N\right)}\gamma_{\left(N-1\right)}+\overset{N-1}{\underset{i=1}{\sum }}\;\gamma_{i}$$

(3) $$\gamma_{i}=\alpha_{1}\times \cdots \times \alpha_{i}=\overset{i}{\underset{j\;=\;1}{\prod }}\;\alpha_{i};\;\alpha_{i}=\frac{k_{p}\left(i-1\right)}{k_{p}\left(i-1\right)+k_{\text{off}}\left(i\right)}$$

(4) $$\delta =\frac{1}{\mu }\frac{k_{p}\left(N-1\right)}{k_{\text{off}}\left(N\right)}\gamma_{\left(N-1\right)}$$

and where *C_T_* is the number of bound receptors or ligands $(=\overset{N}{\underset{i\;=\;0}{\sum }}\;C_{i})$, which is given by

(5) $$\begin{array}{ll} C_{T} & =\frac{T_{T}+P_{T}+∊-\sqrt{{\left(T_{T}+P_{T}+∊\right)}^{2}-4P_{T}T_{T}}}{2};\\ ∊ & =\frac{1}{\mu }\frac{k_{\text{off}}\left(0\right)+k_{p}\left(0\right)}{k_{\text{on}}} \end{array}$$

and being *T_T_* and *P_T_* the total number of receptors and pMHC ligands.

Summarizing, the response can be obtained as a function of *t* by solving numerically the system of ODEs shown in Appendix or analytically under steady-state conditions [equations (1)–(5)], by introducing into them the values of the dissociation and propagation rate constants [*k*_off_(*i*) and *k_p_*(*i*)] for the different types of pMHC ligands engaged in the activation process. If *t* is sufficiently large, the numerical solution response approaches the analytical solution.

### 3.2 Particular Cases

**Figure 2:**
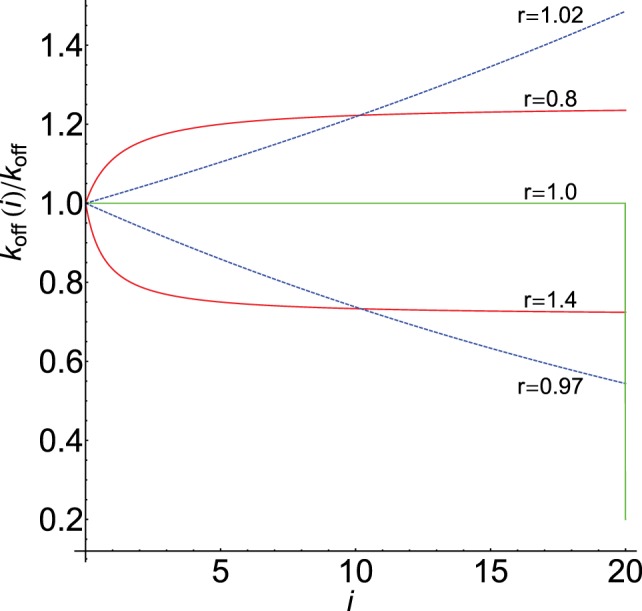
**Dependence of *k*_off_(*i*)/*k*_off_ on *i* for *N* = 20: red [equation (8)], blue [equation (9)]**. Values of *r* shown on the curves. Graph of equation (6) with *c* = 0.2 is in green. The horizontal part of the green plot where *k*_off_(*i*) = *k*_off_ is also the graph of equations (8) and (9) when *r* = 1.

Equations for the T cell response given in the previous section are general and can be applied for any type of function for *k*_off_(*i*) and *k_p_*(*i*). Thus, some particular cases that result from the general solution are as follows:

(a) The simplest situation occurs in the basic KPR when all dissociation and propagation rate constants are the same along the proofreading chain, i.e., *k*_off_(*i*) = *k*_off_ (with *k*_off_ = 1/*τ*) and *k_p_*(*i*) = *k_p_* for all *C_i_* complexes. By inserting these conditions into equations (1)–(5), they are greatly simplified, and after a little algebra we find: *α* = *k_p_*/(*k_p_* + *k*_off_), *C*_0_ = *k*_off_*C_T_*/(*k*_off_ + *k_p_*), *C_i_* = *α^i^C*_0_(1 ≤ *i* < *N*), *C_N_* = *α^N^C_T_*, and where *ϵ* in equation (5) is now *k*_off_/*k*_on_ = *K_D_*, the dissociation constant of the TCR–pMHC complex. These are the well known expressions for the classic KPR mechanism (11, 22), and it provides a test for the correctness of our expressions.
(b) In order to improve the trade-off between sensitivity and specificity, McKeithan (22) modified the basic KPR scheme so that the rate of dissociation for the final productive signal complex *C_N_* is much smaller than for the other complexes in the activation chain:
  (6) $$k_{\text{off}}\left(i\right)=k_{\text{off}}\;\left(i=0,1,\cdots \;,N-1\right),k_{\text{off}}\left(N\right)=c\times k_{\text{off}},c<1.$$
  This modification is a particular case of our general solution, and inserting equation (6) into equations (1)–(5) we find
  (7) $$C_{N}=\frac{\alpha^{N}C_{T}}{\alpha^{N}+c\left(1-\alpha^{N}\right)},\;∊=\frac{cK_{D}}{\alpha^{N}+c\left(1-\alpha^{N}\right)}.$$
  As expected, if *c* = 1, equation (7) simplifies to those of the basic KPR.
(c) As above mentioned, stabilization/destabilization of the *C_i_* complexes in the proofreading chain occurs if *k*_off_(*i*) decreases/increases as *i* → *N*. An appropriate function with these characteristics is
  (8) $$\begin{array}{l} k_{\text{off }}\left(i\right)=\frac{\left(1+i\right)}{\left(1+\mathrm{ri}\right)}\;k_{\text{off }}\;;\;\left\{\begin{array}{c} k_{\text{off }}\left(i\right)\;\text{decreases if }r>1\;\text{approaching}\;k_{\text{off }}∕r\;\text{for}\;i>>1\\ k_{\text{off }}\left(i\right)\;\text{remains}\;\text{constant}=k_{\text{off }}\;\text{if}\;r=1\;\\ k_{\text{off }}\left(i\right)\;\text{increases}\;\text{if}\;r<1\;\text{approaching}\;k_{\text{off }}∕r\;\text{for}\;i>>1 \end{array}\right. \end{array}$$
  where *r* is a parameter that modulates the strength of the activation chain. For antigenic peptides *r* > 1 and those antigens that induce stronger responses will have higher values of *r*. Note that although antigenic peptides have larger *τ* (i.e., smaller *k*_off_) than weak or self-ligands, this initial outcome is largely amplified by equation (8) as activation progresses. Conversely, for self-ligands *r* < 1, which destabilizes the *C_i_* complexes and attenuates the strength of the chain propagation as *i* → *N*. Hence, and as a result of equation (8), the proofreading chain behaves differently for foreign and self-ligands, which gives rise to important and significant consequences in sensitivity and antigen discrimination (see below). Finally, equation (8) shows that for *r* = 1 *k*_off_(*i*) is constant for all the C*_i_* complexes, and we have the same response as in the basic KPR scheme. Another function with similar characteristics to equation (8) is as follows:
  (9) $$\begin{array}{l} k_{\text{off}}\left(i\right)=k_{\text{off}}\times r^{i}\;;\left\{\begin{array}{c} k_{\text{off}}\left(i\right)\;\text{decreases}\;\text{if}\;r<1\;\left(\text{antigenic}\;\text{peptides}\right)\\ k_{\text{off}}\left(i\right)\;\text{remains}\;\text{constant}=k_{\text{off}}\;\text{if}\;r=1\\ k_{\text{off}}\left(i\right)\;\text{increases}\;\text{if}\;r>1\;\left(\text{self}\;\text{ligands}\right) \end{array}\right. \end{array}$$
  The main difference with equation (8) is their behavior when *N > > *1. Under these conditions, equation (9) shows that *k*_off_(*N*) → 0 for antigenic peptides (*r* < 1), while increases without bound for self-ligands (*r* > 1). In other words, for r ≶1 and long proofreading chains, stronger and weaker productive responses will be observed than with equation (8).
  Figure 2 displays the behavior of *k*_off_(*i*)/*k*_off_ computed from equations (8) and (9) for *N* = 20 and different values of *r*. As we show below, this parameter strongly modulates the response through the *k*_off_(*i*)-values for foreign and self-ligands. For comparison, graph of equation (6) and its digital behavior is also displayed (in this case *k*_off_(*i*)/*k*_off_ drops abruptly from 1 to *c* at the end of the chain, i.e., for *i* = *N*).
(d) Regarding the propagation rate constant, *k_p_*(*i*), we have suggested that their values should increase for foreign and decrease for self-ligands. Hence, similar expressions to equations (8) and (9) could also be used for *k_p_*(*i*):
  $$\begin{array}{l} k_{p}\left(i\right)=\left\{\begin{array}{c} \frac{k_{p}\left(1+i\right)}{1+\mathrm{ri}},\;r<1\left(\text{antigenic}\;\text{peptides}\right),\;r>1\;\left(\text{self}\;\text{ligands}\right)\;\left(10\right)\\ k_{p}r^{i},\;r>1\;\left(\text{antigenic}\;\text{peptides}\right),\;r<1\;\left(\text{self}\;\text{ligands}\right)\;\left(11\right) \end{array}\right. \end{array}$$
  where *k_p_* = *k_p_*(0) denotes the rate constant for the transformation *C*_0_ → *C*_1_. As previously, if *r* = 1 we have *k_p_*(*i*) = *k_p_* for all *C_i_* complexes as in the basic KPR.

### 3.3 Discrimination and Sensitivity: Modulation Power of the Activation Chain

In the KPR models proposed so far, specificity and sensitivity between self- and foreign pMHCs are achieved mostly through differences between their dissociation times. In fact, if two pMHCs have the same dissociation time they should provide the same response so that it is not possible to discriminate between these ligands (by assuming that the rest of parameters *k*_on_, *k_p_*, and *N* are also the same). However, in our model, this is not the case since the activation chain can modulate strongly the response in a different way for antigens and self-ligands regardless of their dissociation times. To illustrate this fact, and although antigenic peptides have larger dissociation times than self-ligands, we have considered a situation where two ligands, one supposed to be an antigenic and the other a self-ligand, have the same value of *τ*. If predictions from our model demonstrate that discrimination under these unfavorable conditions would occur, then under more favorable conditions, i.e., when dissociation times for foreign ligands are larger than for self-pMHCs, specificity will be also greatly enhanced.

**Figure 3:**
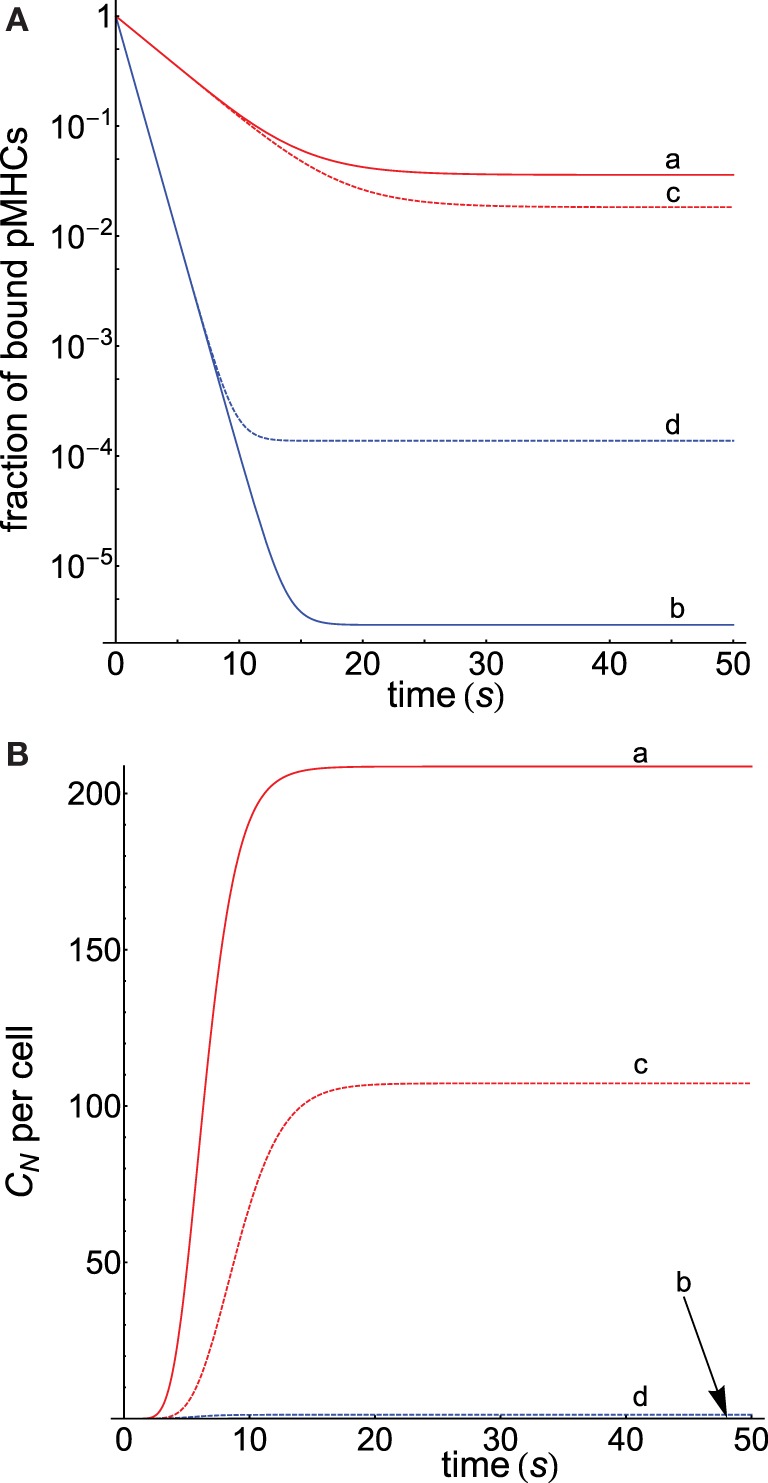
**(A)** Dependence of the fraction of productive pMHCs that remains bound to TCR on time for two kinds of ligands displaying the same *τ* = 2 s, but the first presents a foreign peptide (red curves), while the second contains a self-peptide (blue curves). Computations were performed for *N* = 10 as described in Appendix. *k*_off_(*i*) and *k_p_*(*i*) in the different curves were obtained, respectively, from equations (8) and (10) with the following values of *r*: **(a)** 2.5, 0.5; **(b)** 0.5, 2.5; **(c)** 2.5, 1; **(d)** 0.5, 1. In curves **(c)** and **(d)**, the values of *k_p_*(*i*) = *k_p_* are constant. **(B)** Progress curves of the response *C_N_* as a function of *t* for the cases shown in **(A)**. For curve **b**, practically no response is obtained, and its plot is almost coincident with the *x*-axis.

Since we hypothesized that the activation chain behaves differently for antigens and self-ligands, equations (8) and (10) with appropriate values of *r* for both types of ligands were used to compute the corresponding dissociation and propagation rate constants. The corresponding responses obtained under these conditions are displayed in Figure 3 (computation details are given in Appendix) where Figure 3A shows that, despite both pMHCs having the same dissociation times, the fractions of productive pMHCs remaining bound to the TCR (i.e., *C_N_*/*P_T_*) over time are quite different for the antigenic peptide and for the self-ligand that allows to discriminate between them (curves **a** and **b**). Thus, for a threshold time of 5 s that fraction is 34-fold larger for the foreign ligand (curve **a**) than for the self-ligand (curve **b**), and this value is 1195-fold higher if the threshold is 10 s. In turn, sensitivity is also enhanced, and we find (see Appendix for computation details) that while the foreign antigen requires just a few ligands (<5) to get a productive response, the self-pMHC needs a huge amount of them (>10^9^).

Further insights into this subject are obtained from the behavior of the curves **c** and **d** in Figure 3A, which were obtained proceeding as previously, but with the condition that *k_p_*(*i*) = *k_p_* remains constant along the proofreading chain for both ligands. This eliminates one of the factors that modulate the activation progression so that specificity and sensitivity will be only due to the influence exerted by the different behavior of the dissociation rate constants for foreign and self-ligands. Note that under these conditions, the *k_p_*(*i*)-values in curve **c** are smaller than in curve **a**, while in curve **d** these values are larger than in curve **b**. Hence, this slows down the progression of the activation for the foreign pMHC and speeds up it for the self-ligand and, as a result, curves **c** and **d** are closer than curves **a** and **b**, and the ratio of the fraction of bound pMHCs at 10 s is 566 instead 1195. Also, the required number of ligands to elicit a productive response is 9.3 for the foreign pMHC and 820 for the self-pMHC instead of 5 and >10^9^ ligands previously found when *k_p_*(*i*) was not constant. This shows that, although foreign and self-ligands in this example have the same propagation rate constants, specificity and sensitivity still remain high because of the opposing influences exerted by the dissociation rate constants of both ligands on the proofreading progression. Finally, Figure 3B shows the progression of the productive response *C_N_* as a function of time for the cases displayed in Figure 3A. As expected, and in agreement with results obtained in Figure 3A, curve **a** for the foreign pMHC exhibits the earliest and largest response while, conversely, no appreciable signaling response for the self-pMHC can be detected in curve **b**. Similar considerations apply to the other curves. Also, note that in all curves steady state is reached within tens of seconds.

The above results show that specificity and sensitivity for recognition of foreign and self-ligands result from the modulation power exerted by the activation chain through the values of *k*_off_(*i*) and *k_p_*(*i*) in conjunction with differences between the dissociation times of the ligands. In fact, the modulation power of the activation chain could be as effective that responses overriding differences between dissociation times could occur. This is shown in Table 1 where three different cases have been considered: the self-pMHC has equal, larger, and smaller (this is the normal situation) dissociation time than the foreign pMHC. The ratio of fractions of productive pMHCs remaining bound, and the number of pMHCs required to get a productive response for the three cases have been determined, and included in the table. This demonstrates that even when the dissociation time of the self-pMHC is twice than of the foreign ligand (second row of the table), the value of *C_N_* (foreign pMHC)/*C_N_* (self-pMHC) is still 12.4, while the number of ligands to get a productive response are 4.8 and 758.2 for the foreign and self-pMHCs, respectively.

**Table 1:** **Dependence of specificity and sensitivity on the dissociation times of the foreign and self-pMHCs**.

			Number of ligands^b^	
*τ*/s (foreign pMHC)	*τ*/s (self-pMHC)	Ratio^a^	Foreign pMHC	Self-pMHC
2	2	1194.6	4.8	>10^9^
2	4	12.4	4.8	758.2
4	2	3321.4	2.2	>10^9^

*Values of *k*_off_(*i*) and *k_p_*(*i*) were computed, respectively, from equations (8) and (10) with the following values of *r*: foreign pMHC 2.5 and 0.5; self-pMHC 0.5 and 2.5; *N* = 10*.

*^a^Ratio of fractions of productive pMHCs remaining bound at *t* = 10 s for foreign and self-pMHCs [=*C_N_* (foreign pMHC)/*C_N_* (self-pMHC)]*.

*^b^Number of ligands that produce a productive signaling response under steady-state conditions*.

The length of the proofreading chain is given by the *N*-value, which also contributes greatly to reinforce the activation progression for foreign pMHCs and to its weakening in the case of self-ligands. This is displayed in Table 2 where productive signaling responses for a foreign and self-pMHC with the same dissociation time (2 s) have been determined as a function of *N*. The corresponding response obtained using the standard KPR model for a pMHC ligand with *τ* = 2 s has also been included for comparison. The values of *C_N_* were determined in the presence of a large amount of pMHC ligands (10^7^) to show the different transmission power of the proofreading chain under conditions of pMHC saturation. If we consider that a productive response is attained for *C_N_* ≥ 1 (21), it follows that in the case of self-pMHCs our model predicts the rapid weakening of the activation transmission chain as *N* increases and the break down of the chain progression for *N* ≥ 10. Conversely, the KPR model predicts that a positive response for the self-pMHC will be observed even for *N* = 20. In other words, the different properties of the activation chain against foreign and self-pMHCs cause that the required chain length to achieve discrimination between both types of ligands is much shorter in our model than in the standard KPR scheme, which, in turn, attains the important goal of speeding up the immune response (see Appendix for quantitative details on the speed of the activation chain). This fact, together with results previously displayed in Figure 3 and Table 1, explain why just a few foreign pMHCs are able to trigger a rapid T cell response while huge amount of self-pMHCs are unable to activate the TCRs.

**Table 2:** **Dependence of the productive signaling response *C_N_* on *N***.

	Number of ligands per cell^a^		
*N*	*C_N_* (foreign pMHC)	*C_N_* (self-pMHC)	*C_N_* (KPR model)
4	10447.3	681.7	3946.7
6	8313.9	78.3	1754.1
8	6677.1	8.3	779.6
10	5391.1	0.8	346.5
12	4368.3	8.2 × 10^−2^	154.0
14	3548.4	7.8 × 10^−3^	68.4
16	2888.1	7.3 × 10^−4^	30.4
18	2354.1	6.7 × 10^−5^	13.5
20	1921.3	6.1 × 10^−6^	6.0

*The dissociation time for the three types of pMHCs is 2 s. Other conditions as in Figure 3*.

^a^Values of *C_N_* (productive signaling response) under steady-state conditions; P_T_ = 10^7^, T_T_ = 2 × 10^4^, so that C_N_ is bounded by T_T._

Finally, it has been found that some ligands with short lifetimes can also trigger responses, which have been explained by assuming that these ligands reassociate quickly after unbinding so that their effective binding time is much longer (9). To test if this situation can be also predicted by our model, we have computed *C_N_* for agonists with a short lifetime (*τ* = 0.5 s), and we have found that a response can be triggered with a relatively low number of ligands (*P_T_* = 1000) for chain lengths of up to *N* = 12 (the rest of conditions as in Figure 3). Even if *τ* is as low as 0.1 s, a productive response is still obtained up to *N* = 6, although now a higher number of agonists is required (10^5^).

### 3.4 Comparison with Other Models

In this section, we compare predictions of our model for specificity and sensitivity with the corresponding predictions computed from the standard KPR model, the KPR modified by McKeithan (22) [in this model, the complexes *C*_0_, *C*_1_, …, *C_N−_*_1_ have the same *k*_off_ = 1/*τ* except the final productive *C_N_* complex, which has a much smaller rate dissociation constant, see equations (6) and (7)] and the induced rebinding model recently proposed by Dushek and van der Merwe (21) (in this model the standard KPR scheme is modified to allow for pMHC rebinding). Computation details are given in Appendix.

**Figure 4:**
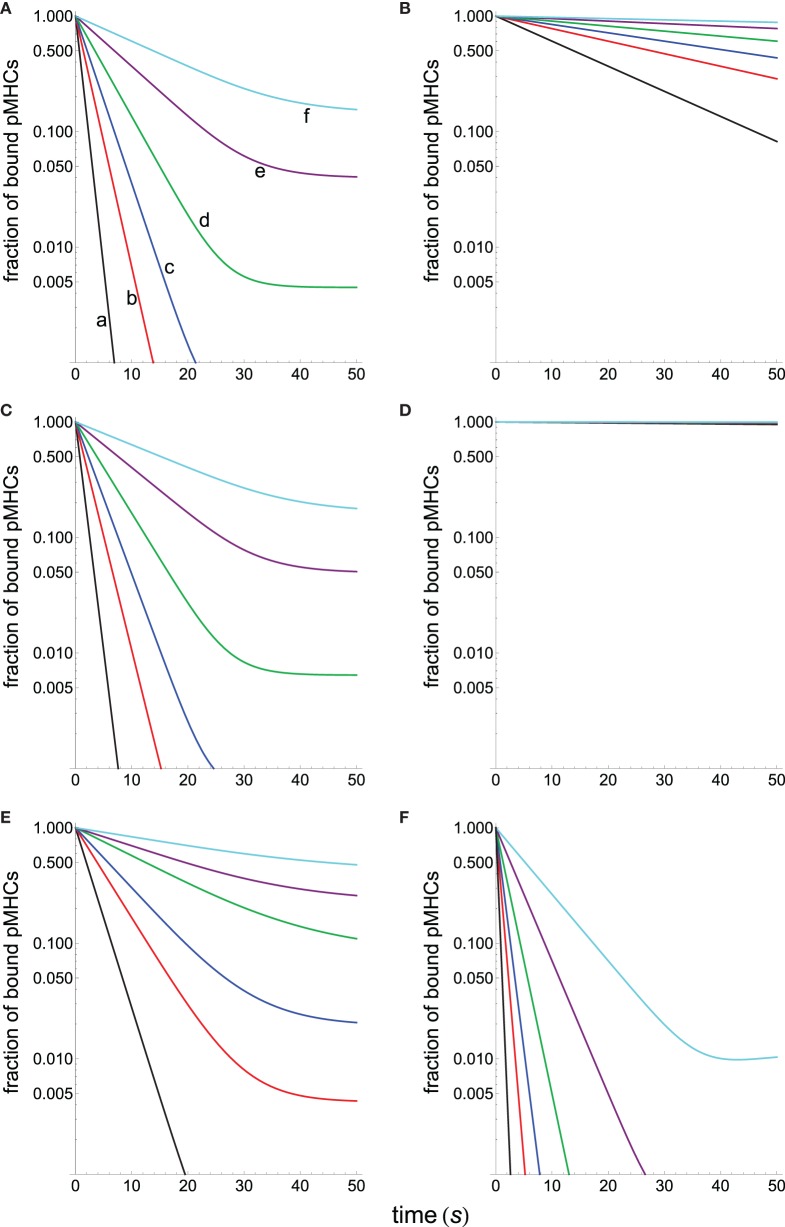
**Dependence of the fraction of productive pMHCs that remains bound to TCR on time for ligands with different dissociation times (s)**. *N* = 20 in all panels except in **(D)** where *N* = 25. **(A)** Basic KPR model: (a) black, *τ* = 1; (b) red, *τ* = 2; (c) blue, *τ* = 3; (d) green, *τ* = 5; (e) purple, *τ* = 10; and (f) cyan, *τ* = 20. Dissociation times in others panels as in **(A)**. **(B)** KPR (McKeithan’s modification). **(C)** Induced rebinding model with *N* = 20. **(D)** Induced rebinding model with *N* = 25. **(E)** Our model: antigenic pMHC with *k*_off_(*i*) and *k_p_*(*i*) computed, respectively, from equations (9) and (11) with the following values of *r*: 0.95 and 1.05. **(F)** Our model: self-pMHC with *k*_off_(*i*) and *k_p_*(*i*) computed as in **(E)** with the following values of *r*: 1.05 and 0.95.

**Figure 5:**
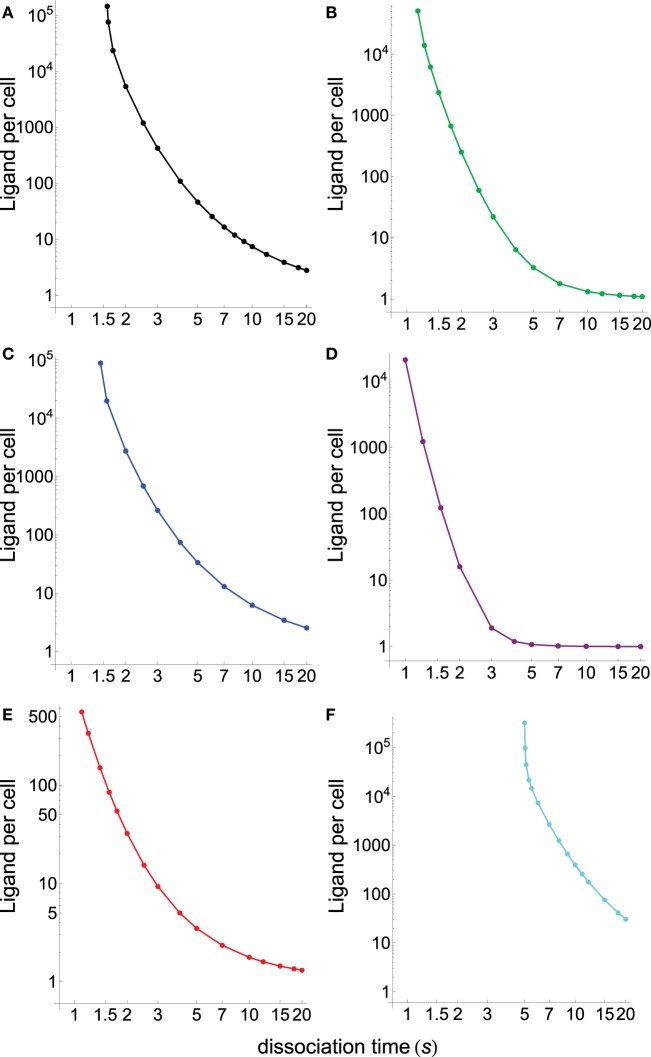
**Number of ligands required to obtain a productive response *C_N_* = 1 calculated under steady-state conditions as a function of their dissociation times for the different models shown in Figure 4**. **(A)** Black, basic KPR model; **(B)** green, KPR (McKeithan’s modification); **(C)** blue, induced rebinding with *N* = 20; **(D)** purple, induced rebinding with *N* = 25; **(E)** red, our model: antigenic pMHC; **(F)** cyan, our model: self-pMHC. Other conditions as in Figure 4.

Figure 4 displays the specificity plots calculated by applying the abovementioned models that show the fraction of productive pMHCs that remains bound to TCR over time for ligands with different dissociation times (s). In turn, Figure 5 displays the corresponding sensitivity plots calculated under steady-state conditions for the same models that appear in Figure 4, i.e., plots in Figure 5 provide the number of ligands required to give a productive response as a function of their dissociation times. From Figures 4 and 5, the following conclusions are drawn:

- As expected, the standard KPR model exhibits a high discrimination capacity (Figure 4A) although the corresponding sensitivity is low (Figure 5A).
- Specificity calculated from the KPR with McKeithan’s modification is largely decreased, while the related sensitivity is enhanced (Figures 4B and 5B).
- Specificity and sensitivity calculated by applying the induced rebinding model with *N* = 20 display large discrimination capacity and low sensitivity (Figures 4C and 5C).
- However, for *N* = 25, specificity and sensitivity plots for the induced rebinding model are quite different. Thus, specificity is almost lost (Figure 4D), while sensitivity is greatly enhanced (Figure 5D).
- That the behavior of the induced rebinding model is quite different for *N* = 20 and *N* = 25 occurred because the rebinding rate constants (*ρ_i_*) are unknown, and it was assumed that for *N* ≤ 20 the *ρ_i_*-values are almost constant $\left(\rho_{i}≃10^{3}\;{\text{s}}^{-1}\right)$ while for N = 25 the *ρ*-value changes abruptly to 10^7^ s^−1^ (21). For these values of *ρ_i_*, induced rebinding has little effect on specificity/sensitivity when *N* = 20 so that their corresponding plots are very similar to those obtained with the standard KPR model [compare **(A)** and **(C)** in Figures 4 and 5]. Conversely, for *N* = 25, the rebinding rate for the productive response is so high (10^7^ s^−1^) that ligands remain trapped for longer period of times within the TCR clusters, which cause a great loss of specificity (Figure 4D) and a large increase in sensitivity (Figure 5D).
- In all the above models, there is a trade-off between specificity and sensitivity [**(A–D)** in Figures 4 and 5].
- Predictions from our model for specificity/sensitivity for a foreign pMHC are shown in Figures 4E and 5E. These plots display a large discrimination capacity as well as a great sensitivity because only a few ligands are necessary to elicit a response even for low values of *τ*.
- Predictions from our model for specificity/sensitivity for a self-pMHC are displayed in **(F)** Figures 4 and 5. Besides the high specificity (Figure 4), it is worth to note the extremely low sensitivity for these kinds of peptides (Figure 5). Thus, for τ ≲5 s the proofreading chain is not established at all, i.e., no productive response is observed, even in the presence of huge amounts of ligands. Furthermore, in the case that self-ligands with larger dissociation times would exist, for example, *τ* = 6 s it follows from Figure 5 that more than 7 × 10^3^ ligands would be required.
- The last two remarks reveal that, in our model, discrimination between foreign and self-pMHCs comes mainly from the different properties of the activation chain for both kinds of peptides. In turn, this is also the reason why such big differences between their respective sensitivities arise regardless of their dissociation times. In other words, if we hypothesized that specificity/sensitivity depend on the transmission chain, then discrimination between foreign and self-pMHCs and their different sensitivities appear as a logical consequence of the fact that the proofreading chain leading to productive response delays and weakens (or even breaks down) for self-pMHCs while reinforces and speeds up for foreign pMHCs as activation progresses. In this case, the trade-off between specificity/sensitivity there would no longer be applicable, what really seems to occur in the adaptive immune response.

## 4 Experimental Considerations

Our assumptions on TCR/pMHC interaction have been modeled by considering that the dissociation and propagation rate constants vary in a different way for foreign and self-ligands along the activation chain. However, as actual values of *k*_off_(*i*) and *k_p_*(*i*) for the *C_i_* complexes are unknown, new challenging experiments to determine these values are necessary to validate or refute our model (which also applies to the supposition that *k*_off_ and *k_p_* do not vary along the proofreading chain on which current models are based). However, measurements of TCR–pMHC binding properties are difficult and, at present, only have been performed for the first complex (*C*_0_) of the proofreading chain (28, 29), although some studies revealed contradictory results (30). Hence, going further ahead in the kinetic proofreading chain is crucial to advance in downstream signaling knowledge although this is experimentally and technically challenging. In this regard, it is a hopeful sign that, even if the *C_i_* complexes in the activation chain would have very similar structures, v.g., conformers with small differences of energy among them, the recently published kinetic analysis of protein conformational changes have shown that conformational states can exhibit different rate constants and affinities (27).

## 5 Conclusion

We show a phenotypic model in which the progression of the proofreading chain occurs quite differently for foreign and self-pMHCs. Our model reveals that the three properties necessary to trigger the TCR/pMHC immune response, namely, speed, specificity, and sensitivity act coordinately so that no signal response will be observed for self-pMHCs, while a large effective response will be obtained with foreign pMHCs. We hypothesize that the different behavior of the activation propagation chain for both types of ligands results from stabilization (foreign pMHCs) and destabilization (self-pMHCs) of the complexes that participate in the proofreading chain. This assumption has been modeled by considering that the dissociation and propagation rate constants vary in a different way for both types of ligands along the activation chain. Deliberately, the model has been formulated as simple as possible to allow that modifications to accomplish for additional features of the immune response can be incorporated. Thus, for example, it has been reported (31) that self-peptides can also been recognized by TCR inducing tonic signals, which could be the result of weaker TCR-induced responses than those elicited by foreign peptides, i.e., that different T cell outcomes are achieved at different TCR signaling thresholds (32). In this regard, we have shown that our model allows modulating the strength and the outcome of the signal response through the parameter *r* involved in the dissociation and propagation rate constants. Nevertheless, like other phenotypic models, no explicit assumptions regarding the mechanisms involved in the stabilization/destabilization of the *C_i_* complexes in the kinetic proofreading chain for foreign and self-ligands have been made, and we expect that future work will elucidate the nature of these processes and their contribution to the immune response.

## Author Contributions

JG and PG-P designed research, performed research, involved in the formulation of the model, analyzed data, and wrote the paper; JJG involved in the formulation of the model and analyzed data.

## Conflict of Interest Statement

The authors declare that the research was conducted in the absence of any commercial or financial relationships that could be construed as a potential conflict of interest.

## Appendix

### A Mathematical Formulation of the Model

The reaction scheme shown in Figure 1 is described by the following system of ordinary differential equations (ODEs):

(A1) $$\frac{\mathrm{dP}}{\mathrm{dt}}=-k_{\text{on}}\mathrm{PT}+\overset{N}{\underset{i=0}{\sum }}\;k_{\text{off}}\left(i\right)C_{i}$$

(A2) $$\frac{\mathrm{dT}}{\mathrm{dt}}=-k_{\text{on}}\mathrm{PT}+\overset{N}{\underset{i=0}{\sum }}\;k_{\text{off}}\left(i\right)C_{i}$$

(A3) $$\frac{dC_{0}}{\mathrm{dt}}=k_{\text{on}}\mathrm{PT}-\left(k_{\text{off}}\left(0\right)+k_{p}\left(0\right)\right)C_{0}$$

(A4) $$\frac{dC_{i}}{\mathrm{dt}}=k_{p}\left(i-1\right)C_{i-1}-\left(k_{\text{off}}\left(i\right)+k_{p}\left(i\right)\right)C_{i};\;1\leq i\leq N-1$$

(A5) $$\frac{dC_{N}}{\mathrm{dt}}=k_{p}\left(N-1\right)C_{N-1}-k_{\text{off}}\left(\text{N}\right)C_{N}$$

where *P* and *T* are the concentrations of free pMHC and TCR, respectively. The parameters that govern the activation chain are *N*, the number of steps leading to the productive signaling complex *C_N_*; the binding rate, *k*_on_; and the dissociation and propagation rates, *k*_off_(*i*) and *k*_p_(*i*). The complexes involved in the activation chain are *C_i_* (*i* = 0, 1, …, *N*) where *C*_0_ is the complex formed by the reversible binding of *P* and *T*, which through a series of chemical modifications leads to *C_N_*. In this regard, we must take into account that for the first step of the binding process (leading to the *C*_0_ complex which is the only one whose kinetic parameters have been experimentally measured) differences in affinity and kinetics between TCRs and self and foreign peptides are not large. This is the reason why the occupancy model (that only considers the *C*_0_ complex) failed to explain the big differences in specificity, sensitivity and speed exhibited by T-cell recognition of self and foreign peptides. This is also the reason why the KPR model assumes that the signaling complex is not *C*_0_ but a *C_N_* complex at the end of the activation chain. However, and although KPR is able to amplify differences in affinity permitting discrimination, KPR by itself is insufficient to explain such big differences.

For simplicity, *k*_off_(0) and *k_p_*(0) are denoted as *k*_off_ (=1/*τ*) and *k_p_* where *τ* is the dissociation time of the complex *C*_0_ and *k_p_* the propagation rate of the step *C*_0_ → *C*_1_. Once the functions *k*_off_(*i*) and *k_p_*(*i*) are provided, for example by using equations (8)–(11) in main text, the system of equations (A1)–(A5) can be solved numerically by applying the appropriate initial conditions. Thus, in order to obtain the fraction of productive pMHCs that remain bound to TCR on time (Figure 3A and different panels in Figure 4) the initial conditions are as follows:

(A6) $$t=0:\;P=0\;,\;T=0\;,\;C_{N}=P_{T}\;,\;C_{i}=0\;\left(i=0,1,\cdots \;,N-1\right)$$

while for the progression curves of the *C_i_* complexes and the *C_N_* response (Figure 3B) we have

(A7) $$t=0:\;P=P_{T}\;,\;T=T_{T}\;,\;C_{i}=0\;\left(i=0,1,\cdots \;,N\right)$$

Finally, we also have the conservation equations,

(A8) $$P_{T}=P+C_{T}\;;\;T_{T}=T+C_{T}\;;\;C_{T}=\overset{N}{\underset{i=0}{\sum }}\;C_{i},$$

where *P_T_* and *T_T_* are the total amount of pMCH and TCR.

The steady-state solution is derived by inserting into the system of ODEs the conditions *dP*/*dt* = *0* (or *dT*/*dt* = 0) and *dC_i_*/*dt* = 0 and by using equation (A8). Thus, equations (1)–(5) in main text are obtained.

### B Computational Details for Figures and Tables in Main Text

#### Figure 3

– Panel A: dependence of the fraction of bound pMHCs (*C_N_*/*P_T_*) on time was computed by solving numerically the system of ODEs equations (A1)–(A5) with the initial conditions [equation (A6)]. Parameters as defined previously with *P_T_* = 1000. Other conditions as given in the caption.
– Panel B: progress curves of *C_N_* computed by solving the system of ODEs with the initial conditions [equation (A7)]. Parameters as defined previously with *P_T_* = 1000. Other conditions as given in the caption.

#### Figure 4

Computations were performed as described previously for Figure 3A. Since both the basic KPR and the KPR modified by McKeithan (22) are particular cases of our general solution, curves for these models (Figures 4A,B) were obtained by solving our system of ODEs with the functions *k*_off_(*i*) and *k_p_*(*i*) corresponding to these models [v.g., in the basic KPR *k*_off_(*i*) = *k*_off_, *k_p_*(*i*) = *k_p_ ∀i*]. For the induced rebinding model (Figures 4C,D) curves were obtained by solving numerically the system of ODEs given by Dushek and van der Merwe (21). Finally, plots for a foreign and a self-pMHC (Figures 4D,F) were obtained by solving our system of ODEs under the conditions given in the caption.

#### Figure 5 and Tables 1 and 2

The number of pMHC ligands required to obtain a productive response can be determined by solving the system of ODEs with the condition that at *t* = *t*_0_ and for a given value of *P_T_* we have *C_N_* = 1. If *t*_0_ is sufficiently large, this value of *P_T_* coincides with that obtained under steady-state conditions, i.e., by solving the equation *δC_T_* = 1 [see equation (1) in main text]. In the induced rebinding model, the above condition is $C_{N}+C_{N}^{*}=1$ (21).

### C Speed of Activation Chain

For a foreign peptide, an estimate of the time required to obtain a productive response is given by

(A9) $$t_{r}=\int_{0}^{N}\;\frac{\mathrm{di}}{k_{p}\left(i\right)}.$$

Thus, for the basic KPR model, we have *t*_r_ = N/*k_p_*, while for our model and using equation (10) in main text we find

(A10) $$t_{r1}=\frac{\mathrm{Nr}+\left(1-r\right)\text{ln}\left(1+N\right)}{k_{p}}\;\left(r<1\right)$$

or, if we choose equation (11)

(A11) $$t_{r2}=\frac{1-r^{-N}}{k_{p}\text{ln}r}\;\left(r>1\right).$$

Hence, the corresponding speedup factors regarding the basic KPR model are given by

(A12) $$f_{1}=\frac{N_{1}}{N_{2}r+\left(1-r\right)\text{ln}\left(1+N_{2}\right)}\;\left(r<1\right)\;;\;f_{2}=\frac{N_{1}\text{ln}r}{1-r^{-N_{2}}}\;\left(r>1\right)$$

where *N*_1_ and *N*_2_ are the number of steps necessary to reach a given level of productive response in the KPR and in our model, respectively. Thus, if we consider that *N*_1_ = *N*_2_ = 20, we have *f*_1_ = 1.736 for *r* = 0.5, i.e., the activation chain speeds up 73.6%, while *f*_2_ = 1.566 for *r* = 1.05. However, because *N*_2_ < *N*_1_ (see Table 2 in main text) these factors will be larger and so, if *N*_1_ = 20 and *N*_2_ = 10, we find *f*_1_ = 3.226 and *f*_2_ = 2.527.
